# Honesty, reliability, and information content of floral signals

**DOI:** 10.1016/j.isci.2023.107093

**Published:** 2023-06-10

**Authors:** Casper J. van der Kooi, Lora Reuvers, Johannes Spaethe

**Affiliations:** 1 Groningen Institute for Evolutionary Life Sciences, University of Groningen, Groningen, the Netherlands; 2 Department of Behavioral Physiology and Sociobiology, University of Würzburg, Würzburg, Germany

**Keywords:** Plant Biology, Plant ecology, Plant evolution

## Abstract

Plants advertise their presence by displaying attractive flowers, which pollinators use to locate a floral reward. Understanding how floral traits scale with reward status lies at the heart of pollination biology, because it connects the different interests of plants and pollinators. Studies on plant phenotype-reward associations often use different terms and concepts, which limits developing a broader synthesis. Here, we present a framework with definitions of the key aspects of plant phenotype-reward associations and provide measures to quantify them across different species and studies. We first distinguish between cues and signals, which are often used interchangeably, but have different meanings and are subject to different selective pressures. We then define honesty, reliability, and information content of floral cues/signals and provide ways to quantify them. Finally, we discuss the ecological and evolutionary factors that determine flower phenotype-reward associations, how context-dependent and temporally variable they are, and highlight promising research directions.

## Graphical abstract

**Figure:**
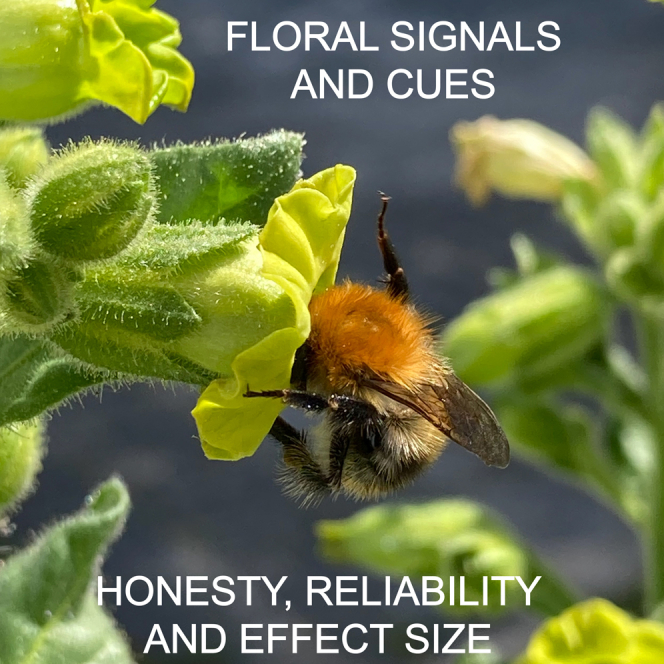

## Introduction

**Figure 1:**
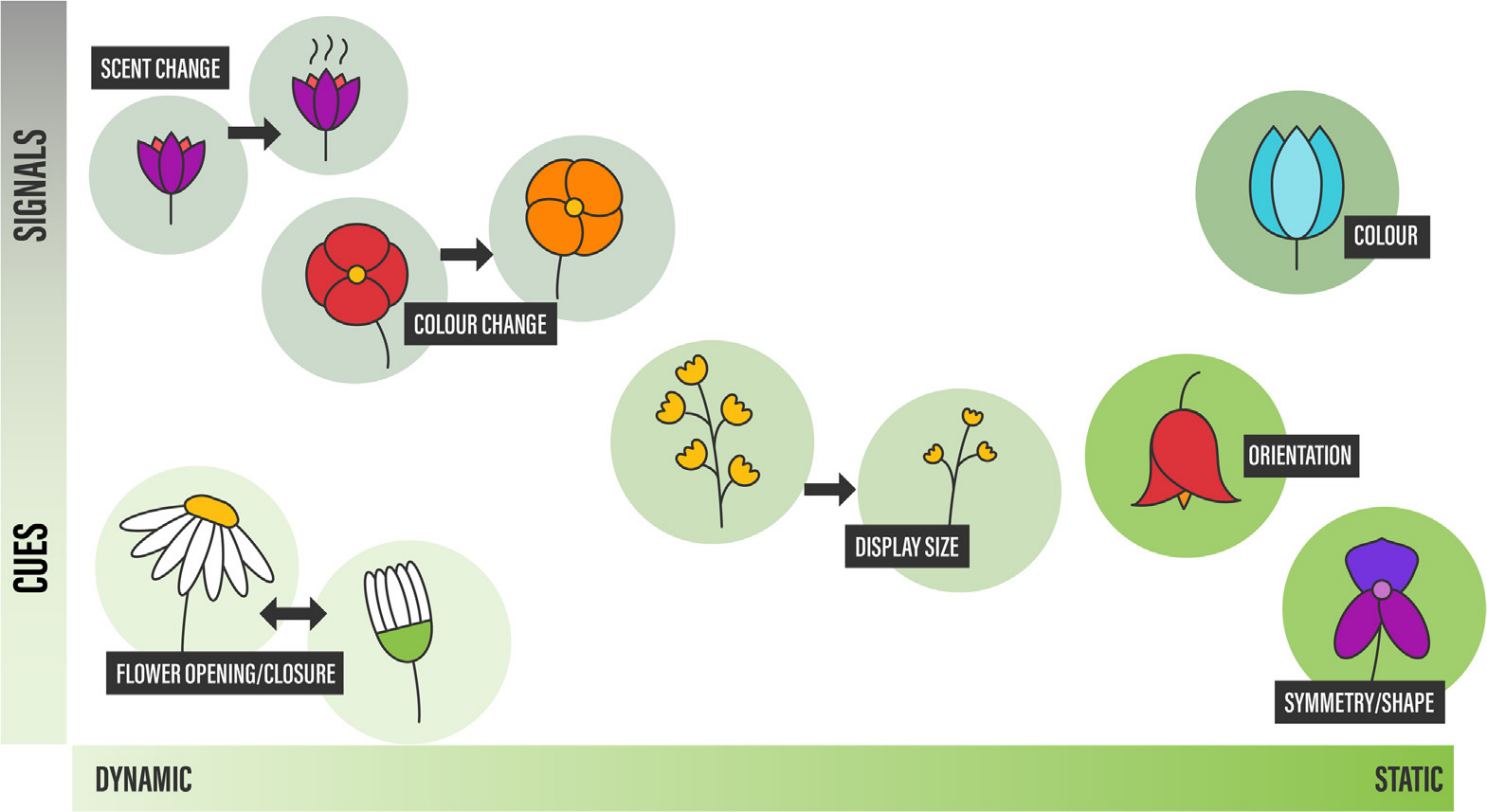
Floral signals, cues, and their dynamicity General features such as floral scent, color, and morphology can aid pollinators in optimizing foraging. Some floral traits can change over short time scales in response to changes in reward availability.

Nearly 90% of flowering plant species are pollinated by animals foraging for floral resources, such as nectar, pollen, and oils.^1^ Plants attract pollinators by presenting floral displays that are often conspicuous in size, shape, color, or scent. Sometimes these floral traits can change during the lifetime of a flower, which may increase the information content of floral signals (Figure 1); for example, flower color can change after pollination. Pollinators detect flowers via different sensory channels, such as vision and olfaction, the sensitivity of which varies between species. Owing to the great diversity in the sensory systems and behavior of pollinators and herbivores,^2^^,^^3^^,^^4^ plants and their floral displays are subjected to different and sometimes contrasting selective pressures. Although sensory systems and behavioral responses of pollinators can co-vary with floral traits, floral signals generally adapt to pollinator sensory systems and not vice versa.^5^^,^^6^^,^^7^^,^^8^ How floral traits convey information to specific pollinators and how those signals may scale with reward quality/quantity is an important question in plant and pollination biology.^9^^,^^10^^,^^11^ That so-called “signal honesty” epitomizes the tension between the often-conflicting interests of plants and pollinators.

To understand how floral traits evolve, how widespread or rare signal honesty is and whether honesty is selected specifically or rather is a by-product, it is essential to use clear definitions, and develop measures to quantify different aspects. In this minireview, we develop a framework that encompasses various aspects of phenotype-reward associations, and we provide ways to quantify them. We first discuss theory and terminology to distinguish floral cues from signals and then provide measures to quantify honesty, reliability, and information content of floral signals and cues. We finally discuss how temporal dynamics may affect phenotype-reward associations, and we highlight promising future research directions.

### Cues versus signals

Floral traits used by pollinators to locate flowers can be signals or cues. The terms signal and cue are often used synonymously, but they differ in meaning. Inspired by Maynard Smith and Harper,^12^ we define a signal as a phenotypic trait that (i) alters the behavior of other organisms, (ii) has originally evolved because of this effect, and (iii) is effective because the receiver’s response has also evolved. In addition, for signals it can generally be said that both signaler and receiver benefit from the signal exchange.^13^ Following that definition, flower color and scent are generally best considered signals, because (i) owing to their distinct smell or color the flower is more conspicuousness to pollinators than without those colors and scent^3^^,^^4^; (ii) for many species the evolution of floral color and scent is considered to be driven by selection that favors high conspicuousness to pollinators^2^^,^^3^^,^^14^^,^^15^; and (iii) pollinators use color and scent to find flowers and their rewards.^16^^,^^17^^,^^18^ An exception to that rule of mutual benefit are rewardless,^19^ deceptive plant species, whereby the plant capitalizes on the pollinator’s preference and there clearly is no benefit for the pollinator.

A cue is a floral trait that pollinators may also use to locate flowers; however cues evolved for reasons other than signaling to pollinators.^13^ A cue’s signaling role can thus often be considered a by-product or epiphenomenon. Zygomorphy (bilateral floral symmetry) and flower orientation are examples of cues. A certain flower symmetry or orientation is shaped by natural selection because it was effective for manipulating pollinator behavior and so increases pollen dispersal^20^^,^^21^ but not to increase attractiveness to pollinators. A change in flower shape need not change the attractiveness to pollinators. Flower orientation can also be determined by environmental effects, such as temperature regulation^22^ (e.g., in east-facing sunflower heads^23^) or protection against rain.^24^ Nevertheless, pollinators may still use shape, symmetry, or orientation to discriminate species. Flower closure is probably also best considered a cue. Although we currently have rather limited knowledge of the factors that drive the evolution of nastic flower movements (opening/closure), current evidence suggests that closure is important to reduce florivory^25^ and to modify flower temperature/humidity.^22^^,^^26^ Pollinators may, however, learn to distinguish closed from open flowers and so increase their foraging efficiency, and it is not impossible that flower opening/closure is a signal in some groups (see below). Another example of a floral cue is CO_2_, which is the result of respiration of flowers. Upon opening, some flowers emit sufficient amounts of CO_2_ to raise the CO_2_-level around the flower up to 200 ppm higher than ambient levels, and pollinators can learn to use it as a cue to locate a floral reward.^27^^,^^28^

The terms signals and cues are often used interchangeably, yet it is important to distinguish the two,^29^ because they arise and are maintained by different mechanistic and evolutionary processes. For signals, the pollinator is a key selective agent. For cues, other selective pressures, such as an abiotic factor or consumption by herbivores, principally determine its origin and maintenance. It is possible, however, that signals evolve from what initially were best considered cues. A cue evolving to become a signal ensues when selection favors increased expression of the trait that attracts pollinators (e.g., stronger scent), and when this increases plant fitness, for example through increased conspecific pollen transfer. Flower closure, for example, may evolve in a way which would justify considering it a signal in specific taxonomic groups. In plants with many simultaneously open flowers (e.g,. trees), closure of pollinated flowers may guide foraging pollinators to open, unpollinated flowers, which would also benefit the pollinators by saving foraging time. This may be the case in, for example, Asteraceae where many species bear large inflorescences and closure can be triggered by pollination.^30^ For flower shape, a similar scenario may occur, because honeybees are known to exhibit an innate preference for certain flower shapes,^31^ meaning they may drive the evolution of flower shape.

It is not always possible to reliably classify a trait as a cue or signal. Whether a trait is a signal or cue can vary between species and contexts. For example, in the majority of cases scent is probably a signal, though it is not hard to imagine a plant that evolved scent to repel herbivores. This scent may have later been co-opted by pollinators, making it a cue rather than a signal.^32^ Another example is flower humidity, which is emitted by leaves and flowers when stomata are open. Recent work suggested that flower humidity can be considered a signal in a hawkmoth-pollinated plant,^33^ though akin to CO_2_ and heat, flower humidity can only be detected at very short distances and is rapidly attenuated by wind. If plants increase flower humidity emission to increase pollinator attraction, it would be a signal. It is furthermore important to consider the phylogenetic signal of the trait of interest. Different pollinator-attracting traits have different evolutionary labilities. Whereas flower color is evolutionarily labile,^4^^,^^34^ flower shape (zygomorphy), for example, typically is more phylogenetically conserved.^35^

Floral display size (the product of the number and size of flowers) is frequently used in studies on “honest signaling”, but it is hard to generally categorize size as a signal or cue. On one hand, flowers evolved to attract pollinators, and visibility to pollinators increases with larger floral displays.^36^ Flower size furthermore correlates with the amount of reward in numerous species,^9^^,^^37^^,^^38^^,^^39^ and key pollinators can learn this species-specific association between size and reward.^40^^,^^41^ On the other hand, both the amount of reward and flower size/number are constrained by various abiotic factors, such as temperature and the availability of water and nutrients. Flower display size therefore generally increases with plant vigor,^42^^,^^43^ so one could argue that floral display size is an allometric by-product of plant vigor, rendering it a cue. Adding to the complexity is that plants are selected for optimal deployment of flowers over a season in terms of availability of mates and likelihood of producing inbred offspring of reduced quality.^24^^,^^29^

Finally, whether a floral trait can be considered as a cue or signal also depends on the flower’s visitors. A plant with red flowers and without reflection in the ultraviolet may attract hummingbirds as a legitimate pollinator for which red is a salient color.^14^ In some cases, however, bees, for which red colors without UV are not attractive, might use the red color as cue to locate and rob the flower (examples mentioned in Chittka and Waser^44^). We must furthermore bear in mind that pollinators, especially bees, can be trained to distinguish all sorts of stimuli, including ecologically irrelevant ones. For example, bees can distinguish between Monet and Picasso paintings^45^ as well as between different human faces.^46^ That ability to learn a variety of tasks attests to the remarkable cognitive abilities of bees, but these abilities are obviously not necessarily ecologically relevant. In summary, evidence to suggest that a trait can be considered a signal must span beyond the fact that pollinators can learn that trait to locate a flower.

### Honesty, reliability, and effect size

**Figure 2:**
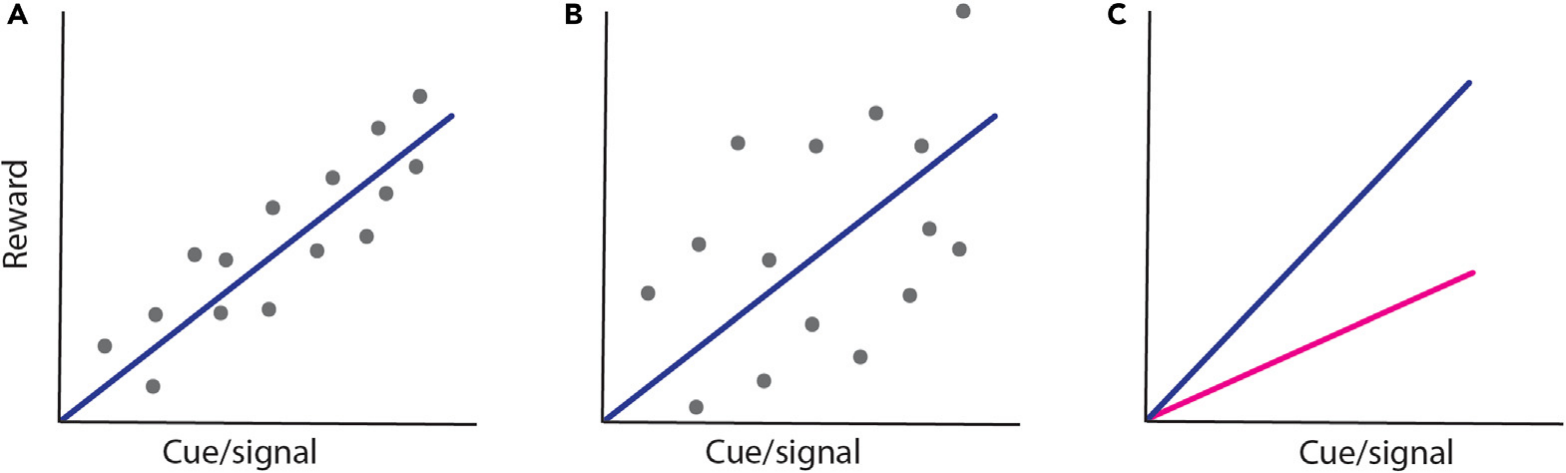
Honesty, reliability, and information content of floral cues and signals (A) The cue/signal is honest when it is significantly correlated with reward status. (B) The variation (residuals) is a proxy for the reliability of the cue/signal (reliability is high in A and low in B) and plays an important role in determining whether a trait is honest or not. (C) The slope is a proxy for the information content of the cue/signal and could be taken as a measure of the effect size (blue line indicates strong effect size, magenta line low effect size).

The terms “honesty” and “reliability” are commonly and sometimes synonymously used to describe plant phenotype-reward associations, but they do not necessarily have the same meaning. It is furthermore not always clear how to quantify the biological effect size of a signal or cue. Inspired by studies on animal behavior, ^e.g.,^^47^^,^^48^ we here propose three different ways by which plant phenotype-reward associations can be characterized in an ecologically meaningful way (Figure 2).

The “honesty” of a phenotype-reward association is determined by whether the phenotype (be it a cue or a signal) is significantly correlated with reward status. The honesty of the phenotypic trait will be primarily determined by the mechanistic coupling of the cue/signal and the reward status. For example, if scent emission is physiologically coupled to nectar synthesis, the relationship is likely to be honest. It is of course important to know whether the pollinator can perceive the signal or cue, and, if so, whether the pollinator can learn the association. For example, if scent emission is coupled with the amount of reward, but the pollinator is not equipped with an olfactory system to detect the scent, the association is not ecologically meaningful. In that case, the association is almost certainly not driven by pollinators. From a pollinator’s point of view, the honesty is important for the animal’s foraging decisions, i.e., to decide what plant species to visit, for how long, and how often. When the reward itself emits a signal, for example in species with very conspicuous nectar or pollen,^49^ the honesty can be estimated directly from the reward’s size or volume. More common are indirect signals/cues,^9^^,^^10^ where the color or scent of the corolla, petals, or bract correlate with a reward that is hidden inside the flower.

We classify the “reliability” of a cue/signal as the variance of that correlation (e.g., measured by the residuals), meaning that it depends on the level of variation in reward amount/quality. Reliability is an important aspect of phenotype-reward associations, because a trait that provides high honesty but low reliability does not allow efficient reward exploitation. Indeed, two signal-reward relationships can both be significant but have very different underlying data distributions, and so both signals are honest but strongly differ in reliability (Figures 2A and 2B). Standing variation and plasticity in reward synthesis and cue/signal production are key components of reliability.

The “information content”, strength, or effect size of a cue/signal is probably best estimated by the slope of the curve that represents the phenotype-reward association (Figure 2C). Assuming the two correlations plotted in Figure 2C are equally significant (honest) and have identical variance (reliability), a certain change in the cue/signal will yield an about 2-fold change in reward for the dark blue as compared to magenta line (the slopes should be standardized for comparative purposes).

It is in the pollinator’s interest to gain as much reward as possible and to easily discriminate between high- and low-rewarding flowers. Therefore, one would expect pollinator-mediated selection for low variation (i.e., high reliability) and strong effect sizes, because it enables fast and efficient reward exploitation.^50^ Plants, on the other hand, may have physiological constraints that limit their phenotypic reliability, and providing high reliability and effect size per se are not in their prime interest. We will now discuss a few case studies to illustrate our delineation of honesty, reliability, and information content at the interspecific as well as intraspecific level.

### Putting honesty, reliability, and information content into context

Studies on phenotype-reward associations in pollination can be roughly categorized into two groups: those that focus on associations at the community-wide scale versus those that study associations in one or few species. Community-wide studies can reveal broad ecological patterns, include multiple species that share a generalist pollinator, and identify elements that are shared among species or networks. Taxon-specific studies will exchange breadth for detail, and can so classify standing intraspecific variation, illuminate the importance of trait variation in the interaction, and potentially uncover what evolutionary factors drove the interaction’s extant phenotype. As an example, in a certain community, plants with blue flowers provide nectar that is easily accessible for bees, whereas red-flowered plants in the same community have specialized on bird pollination and physically prevent bees from taking nectar. For bees, the blue color then is an honest indicator of reward at the community level. Blueness may also be an indicator of species’ reward status *at a specific moment*, for example if the blue color fades after the flower is depleted from nectar. If that is the case, blueness is a “real time” honest indicator of reward status at the species level.

The taxonomic level at which an association is studied is paramount for understanding underlying dynamics, and the results on the degree of honesty, reliability, and information content can be fundamentally different. For example, if at the population level, scent emission is significantly correlated with nectar quality, scent is an honest trait at the population level. If, however, a species in that population cheats and produces scent but no nectar, the association is not honest for that species. Community-wide studies may thus reveal more information on the ecological facilitation of interactions, whereas taxon-specific studies may be particularly revelatory about individual fitness consequences.

Several recent studies investigated the association between floral visual signals and reward at the community-wide scale. Ortiz et al.^51^ studied 98 Mediterranean bee-pollinated plant species and found that at the community level, flower size is the most informative cue for the amount of pollen and nectar. There was, however, enormous variation in the reliability of flower size as a proxy for different rewards. Flower size is a more reliable indicator for pollen volume than for sugar mass. Streinzer and colleagues,^52^ sampling 105 plant species in an alpine flower community, found that flower color category (hue) is an honest, though unreliable indicator of nectar quantity. Some types of colors (blue/purple) produce slightly more nectar than others, but the difference is small and the variation within color categories is large.

The evidence regarding flower color contrast to the (green) background and nectar status at the community level is ambiguous. Color contrast is a widely accepted and broadly applicable measure for visual conspicuousness of flowers and is calculated using vision models.^53^ Ortiz et al.^51^ found a positive but very weak correlation between color contrast and nectar concentration, and Shrestha et al.^54^ found no such association for bee-pollinated flowers in an Australian community. Streinzer et al.^52^ observed a negative association between color contrast and the amount of nectar available. Such contradictory results may arise from differences in the applied statistics or phylogenetic composition of the included taxa, and currently preclude drawing a general conclusion.

Comparing the information content (slope) between different cues/signals and rewards can provide interesting results when possible. When the scales are the same but two curves have different slopes (Figure 2C), it means that one cue/signal has higher information content than the other. When the axes ranges between two comparisons are very different—which is likely to happen when different floral traits are compared—differences in slope are hard to interpret in an ecologically meaningful way, which is why scales should be standardized. As an example, in the study by Ortiz et al.,^51^ flower size is significantly correlated with both number of pollen grains and pollen volume, which is the number and size of pollen combined. The slopes between the correlations differ enormously (6605 vs. 0.23), but when considering the whole data distribution (or when normalizing the data), the slopes are about the same (see Figure 3 in Ortiz et al.^51^), meaning that the overall relationship is similar. A similar case is found in *Penstemon digitalis*. Burdon et al.^55^ found a significant correlation between scent (linalool) emission and both nectar volume and sugar amount, and when the two correlations are plotted, the relationship is roughly the same (see Figure 1 in Burdon et al.^55^), likely because the total amount of nectar and sugar are correlated. A convincing example of differing slopes is found in the study by Benitez-Vieyra et al.^56^ who compared visual floral cues (flower lip area and tube area) for bee versus bird pollinated *Salvia* species. Bees use the lip as visual cue and birds use the tube area as a visual cue. They thus found that for bee-flowers, there is a stronger correlation between lip area and nectar than between corolla area and nectar, and vice versa for birds.^56^ In the seminal work by Stanton and Preston,^38^ it was found that different *Raphanus sativus* populations have different effect sizes for flower size and reward, although there were differences in significance (honesty) too. For *Brassica rapa*, different scent molecules and corolla size may have different effect sizes for the amount of pollen and nectar sugar content.^11^

Floral display size might be the most studied cue in the context of honest signaling, and in many cases, corolla, petal, or bract size correlate with reward.^9^^,^^11^^,^^38^^,^^57^^,^^58^ It is not always clear up to what extent these associations are by-products of plant allometry and whether plants also maintain the association when water or nutrients are limited (see aforementioned, “Honesty, reliability and effect size”). Increasing the number of flowers, and thereby floral display size, generally increases the visibility to pollinators and correlates with an overall higher reward, but also bears an important ecological cost. The number of flowers strongly correlates with the degree of self-pollination via pollinators, i.e., geitonogamous self-pollination.^59^^,^^60^^,^^61^ For example, experiments with *Mimulus ringens* showed that each additional seed sired via geitonogamous self-pollination was associated with a loss of almost ten seeds sired through cross-pollination.^61^ Rewarding plants may thus benefit from a comparatively moderate reliability of their phenotype-reward association, because it will discourage pollinators to stay on the same plant, and hence reduces the incidence of geitonogamy and increases the likelihood of pollinators traveling further, which both increases gene flow.^60^^,^^62^

### Temporal dynamics of phenotype-reward associations

Floral cues, signals and rewards commonly change during a flower’s lifetime. Understanding the temporal dynamics of phenotype-reward associations is a crucial component for evaluating the honesty and reliability of cues and signals, because trait dynamicity can mask or exaggerate existing correlations. When a pollinator visits a flower and harvests (part of) the reward, a subsequent pollinator encounters a (partially) emptied flower. The duration of such a temporal mismatch between cue/signal and reward has consequences for the honesty and reliability of the cue/signal. It need not always be maladaptive for the plant to offer no reward for a short while, because pollinators sometimes only notice there is no reward when they have probed the flower and so some pollen transfer may have occurred already. Yet, it is likely that pollinators will learn when a phenotype is unrewarding, which would lead them to avoid visiting that species. Pollen transfer may decrease when the duration of rewardlessness outweighs the pollinator’s tolerance for rewardlessness, because then the pollinator may decide to switch to another plant species.

The availability of rewards is mainly determined by two processes: reward production by the plant versus depletion by flower visitors. Reward synthesis is temporally and spatially variable, for two reasons. The production rate of rewards can change over time with circadian rhythms, seasons, flower age, or in response to pollination, and it can depend on geographic location (e.g., caused by abiotic effects such as water or nutrient availability), sexual morph, and resource availability.^10^ The condition of the plant seems more important for the production of nectar than for the production of pollen,^37^ albeit we know little about the temporal patterns of pollen release. Pollen release strategies can vary, at least between species and pollination systems,^63^ but within species pollen dosing is presumably less flexible than nectar production. Whether the reward production rate is upregulated after depletion, and, if so, at what speed the reward amount is refilled, has implications for the temporary reliability of a plant’s signals. Nectar production could be upregulated after depletion, or at least in response to flower visits, though higher production rate may lead to changes in chemical composition and thereby affect quality.^64^ A recent paper on a bat-pollinated shrub showed that nectar production is positively correlated with the amount of pollen in the anthers and negatively correlated with pollen on stigmas,^65^ suggesting that nectar availability is a way to manipulate bat visitation behavior.

Nectar standing crop and pollen availability depend on the flower’s visitation history. The plant-pollinator interaction itself can thus modulate the signal’s degree of reliability. As a result, a flower’s depletion rate depends on the plant community, the abundance of rewarding flowers and various aspects of the pollinator community, such as pollinator abundance, foraging strategies, patterns of activity, and removal efficacy. An important result can be that flowers that offer a high reward and corresponding signal are increasingly emptied by visitors. Increased visitation then automatically leads to reduced reward availability, which would decrease the reliability of the signal. In a recent study in the Austrian Alps, it was found that purple/blue flowers on average produce slightly more nectar compared to other flower colors, but when the nectar-standing crop was measured under natural conditions where bees freely visited flowers in the field, this difference in nectar production between colors disappeared.^52^ This indicates that floral color is an honest signal for nectar content but has only a weak reliability, because pollinators learn it and empty these flowers frequently under natural conditions and so lowered the reliability.

Finally, the behavioral response of pollinators increases when the cue/signal differences increases,^38^^,^^50^^,^^55^ because animals usually tend to generalize among signals when they become too similar and so are difficult to distinguish. For example, some food deceptive orchids exhibit marked intraspecific flower color variation, and the variation overlaps with one or more model species.^66^ Therefore, despite their on average distinct color appearance, pollinators do not discriminate between orchid and models due to the probably high costs of missing nectar or pollen rewards because of false discrimination against rewarding flowers (but see ref. ^67^ for an alternative case in which intraspecific color variation is a consequence of relaxed selection by pollinators). The costs decrease with increasing signal differences, which is predicted by signal detection theory.^68^ The use of a complex flower signal that encompasses several sensory modalities like color, scent, and shape can reduce uncertainty about the correct decision,^69^^,^^70^ but in several cases, pollinators only focus on one sensory modality such as color and ignore others such as odor.^66^

### Concluding remarks

The association of floral phenotypes and rewards is of high relevance in plant-pollinator interactions, but due to the multidimensionality of floral signals, rewards and pollinator perception, it can be hard to disentangle underlying processes. The terms signal and cue are often used interchangeably, but have different meanings and as traits they are subject to different selective pressures. Signals are floral traits that primarily evolved because they were selected for by pollinators, which use them to locate floral rewards. Cues are floral traits that primarily evolved because they were selected for by selective agents other than pollinators and their sensory apparatus, although pollinators may still use cues to locate flowers. For multiple plant taxa, there is convincing evidence that floral signals/cues are correlated with reward status, i.e., the traits are “honest”. These signals/cues can be static (e.g., a certain flower color or shape) as well as dynamic, such as color or scent that changes upon pollination. In addition, the reward availability is usually also dynamic, depending on the visitation rate by and removal efficiency of pollinators, and reward synthesis.

Many ecological studies investigate the relationship (“honesty”) between the signals/cues of flowers and their rewards. However, honest correlations can be masked—for both the pollinator and scientist—by high variation in reward availability, due to the complex interplay of differences in reward synthesis and depletion by visitors. Reliability of the cue/signal is thus expected to be as important as honesty, and reliability is probably extra important in environments that are comparatively “poor” in rewards.^48^ The information content of a trait might be particularly important for a pollinator when it decides which floral traits it should consider when choosing the most profitable flowers.

We identified several conspicuous knowledge gaps. There is a clear paucity of data regarding the nutritional properties of rewards, particularly that of pollen. Whereas a metric for sugar composition may reasonably adequately capture nectar quality, pollen generally contains many types of nutrients,^71^^,^^72^^,^^73^ making it difficult to quantify. It is furthermore important that for any type of signal/cue or reward both mean values as well as variance in trait means are estimated. Mean values can illuminate whether there are phenotype-reward correlations, i.e., whether there is honesty, but obtaining measures of variance enables estimating the reliability of the correlations. Combining measures of reward availability with and without pollinator access subsequently provides an estimate of how plant-pollinator dynamics influence the correlation between reward and floral signal/cue in the field.
